# Cytokine Profiles as Molecular Markers Associated with Physical Exercise and Insulin Therapy in Patients with Type 2 Diabetes Mellitus

**DOI:** 10.3390/cimb48070674

**Published:** 2026-06-30

**Authors:** Danielle Cristina Honorio França, Alan Cardec Barbosa, Elton Brito Ribeiro, Anibal Monteiro de Magalhães Neto, Emanuelle Carolina Honorio França, Maraisa Delmult Borges, Patrícia Gelli Feres de Marchi, Adenilda Cristina Honorio-França, Eduardo Luzía França

**Affiliations:** 1 Health Sciences Institute, Federal University of Mato Grosso, Sinop 78557-287, MT, Brazil; danielle.franca@ufmt.br (D.C.H.F.); eltonbr8@gmail.com (E.B.R.); 2 Graduate Program in Basic and Applied Immunology and Parasitology, Federal University of Mato Grosso, Barra do Garças 78600-000, MT, Brazil; acb.alan@hotmail.com (A.C.B.); professoranibal@yahoo.com.br (A.M.d.M.N.); patricia.marchi@ufmt.br (P.G.F.d.M.); 3 Institute of Biological and Health Science, Federal University of Mato Grosso, Barra do Garças 78600-000, MT, Brazil; emanuellefranca57@gmail.com (E.C.H.F.); maraisadelmut@gmail.com (M.D.B.)

**Keywords:** type 2 diabetes mellitus, cytokine profile, immunometabolic regulation, physical exercise, insulin therapy

## Abstract

Background: Type 2 diabetes mellitus is characterized by chronic low-grade inflammation, immune dysregulation, and metabolic impairment. This study investigated cytokine profiles associated with physical exercise and insulin therapy in patients with Type 2 diabetes mellitus. Methods: Blood samples were collected from 51 volunteers to evaluate metabolic parameters and cytokine concentrations. According to glycemic status and insulin use, participants were classified into non-diabetic, non-insulin-dependent Type 2 Diabetes Mellitus, and insulin-dependent Type 2 Diabetes Mellitus groups. Results: Physically active individuals with non-insulin-dependent Type 2 Diabetes Mellitus exhibited increased IL-4, IL-6, and IL-10 levels, suggesting enhanced immunoregulatory and anti-inflammatory responses. Physically active patients with insulin-dependent Type 2 Diabetes Mellitus showed elevated IL-17 concentrations. In contrast, sedentary individuals with insulin-dependent Type 2 Diabetes Mellitus exhibited higher TNF-α levels, indicating a more pronounced proinflammatory profile. IFN-γ concentrations were significantly higher in patients with insulin-dependent Type 2 Diabetes Mellitus, regardless of exercise status. Correlation analyses demonstrated distinct cytokinemetabolic interaction patterns according to metabolic condition and physical exercise. Conclusion: Cytokines can serve as molecular markers of immunometabolic responses associated with physical exercise and insulin therapy in Type 2 Diabetes Mellitus, reflecting alterations in systemic inflammatory regulation and immune–metabolic crosstalk related to glycemic adaptation.

## 1. Introduction

Type 2 diabetes mellitus (T2DM) is a progressive metabolic disorder characterized by chronic hyperglycemia resulting from inadequate pancreatic β-cell insulin secretion in the context of insulin resistance, leading to defects in insulin secretion, insulin action, or both [1,2]. The global incidence and burden of diabetes mellitus have increased substantially in recent decades, making T2DM the most prevalent form of the disease, accounting for approximately 90–95% of all cases [3]. Persistent hyperglycemia induces significant alterations in carbohydrate, lipid, and protein metabolism [4], with significant clinical consequences for affected individuals.

Several pathogenic processes are involved in the development and progression of diabetes. In addition to insulin resistance and β-cell dysfunction, T2DM is increasingly understood as a condition characterized by altered inter-organ metabolic crosstalk, mitochondrial dysfunction, oxidative stress, endoplasmic reticulum stress, and local and systemic inflammation [5,6]. Studies have shown changes in the immune response in diabetic patients [7], which may be associated with increased susceptibility to infections and with the chronic low-grade inflammatory state characteristic of the disease. In addition to hyperglycemia, T2DM is frequently associated with other metabolic disturbances, including dyslipidemia, hypertension, and obesity [8]. Growing evidence suggests that chronic low-grade inflammation is a key mechanism linking these metabolic abnormalities to insulin resistance, disease progression, and the development of complications [9,10].

Biochemical, hormonal, and immunological mediators regulate inflammation. Among these, cytokines are soluble proteins that may or may not be glycosylated and perform multiple metabolic and immunological functions, including cell activation, proliferation, differentiation, and mediation of inflammatory responses. Cytokines can also induce or inhibit the production of other cytokines and may exert endocrine-like effects [11].

The response and production of inflammatory cytokines in patients with diabetes have been identified as possible pathophysiological mechanisms. Some studies suggest that inflammation and hyperglycemia may be related to changes in plasma cytokine levels [12,13]. Moreover, chronic low-grade inflammation and cytokine dysregulation are recognized contributors to insulin resistance and the progression of T2DM [14,15]. Cytokines are important mediators of this inflammatory response and have been implicated in regulating glucose metabolism, insulin signaling, and immune function [10,14]. Therefore, evaluating cytokine profiles may provide valuable insights into the immunometabolic alterations associated with T2DM and the effects of interventions such as physical activity and insulin therapy. This evidence supports the concept that T2DM-associated inflammation involves immune–metabolic interactions, including activation of innate and adaptive immune pathways and cytokine-mediated impairment of insulin signaling.

Although the precise role of these cytokines in T2DM has been partially elucidated, the inflammatory process induced by metabolic imbalance and insulin resistance may accelerate disease progression and complications. Thus, immune system homeostasis is essential to limit inflammatory mechanisms, particularly through the balance between proinflammatory Th1 and Th17 responses and anti-inflammatory or regulatory Th2-associated responses. Diabetes appears to alter the distribution and activity of these immune cell subsets, with increased Th1 and Th17 responses and reduced Th2 activity, consequently modifying the cytokines produced by these cells [16]. The therapeutic approach to T2DM requires multidisciplinary management, including nutritional guidance [17], pharmacological treatment [18], and regular physical exercise [19,20]. Current diabetes care recommendations continue to emphasize lifestyle intervention, individualized pharmacological therapy, and physical exercise as central components of T2DM management [2,18].

Regular physical exercise is one of the recommended strategies for individuals with T2DM because it improves glycemic control, insulin sensitivity, cardiovascular risk, and metabolic health. In association with dietary re-education, the use of hypoglycemic drugs, and, in more advanced cases, insulin therapy, exercise constitutes one of the main pillars of diabetes treatment [21]. Evidence from randomized trials and meta-analyses indicates that exercise can reduce inflammatory mediators, including CRP, TNF-α, and IL-6, in patients with T2DM [22]. Meta-analytic data also suggest that resistance exercise training may improve inflammatory and metabolic parameters in adults with T2DM [23].

The increase in anti-inflammatory cytokines during physical exercise may contribute to the attenuation of proinflammatory responses related to skeletal muscle stress and systemic metabolic inflammation. This mechanism can also inhibit the production of proinflammatory cytokines and help prevent or reduce diabetes-related complications, cardiovascular diseases, and metabolic syndrome [24,25,26].

Although regular physical exercise can contribute to glycemic control and modulate cytokine-mediated inflammatory processes in diabetic patients, the combined effects of physical exercise and insulin therapy on cytokine profiles remain only partially understood. Additionally, it is important to evaluate how therapeutic interventions, including insulin and other antidiabetic drugs, modulate immune–metabolic responses in metabolic diseases. Thus, the present study aimed to assess the influence of insulin therapy and regular physical exercise on the blood cytokine profile of patients with T2DM.

## 2. Materials and Methods

### 2.1. Subjects

This study used a convenience sample composed of eligible volunteers recruited during the study period. A total of 51 volunteers were recruited from the Health System Program of Barra do Garças, Mato Grosso, Brazil. All participants provided written informed consent before enrollment. The local Research Ethics Committee approved the study. Participants were allocated to a non-diabetic group (ND; n = 15) and a Type 2 Diabetes Mellitus group (T2DM; n = 36) based on a prior diagnosis of T2DM per the American Diabetes Association (ADA) diagnostic criteria [27]. To further characterize glycemic status, serum glucose and glycated hemoglobin (HbA1c) levels were evaluated in all participants.

Patients with T2DM received standard clinical management, including pharmacological and non-pharmacological interventions such as dietary modification. Patients were stratified into non-insulin-treated T2DM (NI-T2DM; n = 19), who received oral hypoglycemic agents, and insulin-treated T2DM (I-T2DM; n = 17), who received insulin therapy. In addition, participants were characterized by body mass index and level of physical activity. Individuals who performed regular aerobic exercise, including treadmill walking or running, for at least 6 months, with sessions lasting 45–60 min at least four times per week, were classified as physically active.

Inclusion criteria comprised adults aged 18–40 years who agreed to participate in the study and provided written informed consent. Participants with T2DM had a previous diagnosis of Type 2 Diabetes Mellitus according to established diagnostic criteria, whereas non-diabetic participants had no history of diabetes. In addition, all participants were required to present negative serology for syphilis, HIV, and hepatitis viruses. Exclusion criteria included Type 1 Diabetes Mellitus and other conditions associated with hyperglycemia; current use of corticosteroids or other medications known to affect glycemic control; use of immunosuppressive agents or chemotherapy within the 30 days preceding enrollment; previous diagnosis of autoimmune diseases; and failure to provide written informed consent.

### 2.2. Blood Sampling

Approximately 10 mL of peripheral blood was collected from each participant into anticoagulant-free tubes. Samples were centrifuged at 160× *g* for 15 min to obtain serum. The samples were aliquoted and stored at −80 °C to preserve sample integrity until analysis. A single blood sample was collected from each participant, and all available samples were analyzed under the same experimental conditions to minimize assay variability.

### 2.3. Glucose Determination

Serum glucose concentrations were determined using an enzymatic colorimetric method as previously described by Morceli et al. [4]. Briefly, 20 μL of serum or glucose standard solution [100 mg/dL; BioTécnica^®^, Varginha, MG, Brazil was added to 2.0 mL of phosphate-buffered solution (0.05 M, pH 7.45) containing aminoantipyrine (0.03 mM), sodium p-hydroxybenzoate (15 mM), glucose oxidase (12 kU/L), and peroxidase (0.8 kU/L). According to the manufacturer, the intra-assay and inter-assay coefficients of variation for serum samples ranged from 0.7% to 1.1%. The mixtures were homogenized and incubated at 37 °C for 5 min. Absorbance was measured at 510 nm using a spectrophotometer.

### 2.4. Quantification of Cytokines

Serum samples were thawed immediately before analysis, and cytokine concentrations, including IL-2, IL-4, IL-6, IL-10, IL-17, TNF-α, and IFN-γ, were quantified using a cytometric bead array (CBA) assay (Becton, Dickinson and Company (BD Biosciences), San Jose, CA, USA) according to the manufacturer’s instructions. Data acquisition was performed using a FACSCalibur flow cytometer (Becton, Dickinson and Company (BD Biosciences), San Jose, CA, USA), and analyses were conducted using FCAP Array software version 1.0 (Becton, Dickinson and Company (BD Biosciences), San Jose, CA, USA).

### 2.5. Statistical Analysis

Data are presented as mean ± standard deviation (SD). Normality was assessed using the D’Agostino normality test. Comparisons between groups were performed using one-way analysis of variance (ANOVA), followed by Tukey’s post hoc test. Correlations between blood glucose levels and cytokine concentrations were evaluated using Pearson’s correlation coefficient. Statistical significance was set at *p* < 0.05.

## 3. Results

### 3.1. Subsection

Clinical characteristics of the study population are presented in Table 1. Serum glucose concentrations were higher in patients with T2DM than in those with ND. Among patients with T2DM (n = 36), 17 were classified as insulin-treated (I-T2DM) and 19 as non-insulin-treated (NI-T2DM). Regular physical exercise was reported by 7 of 15 ND individuals (46.7%) and 16 of 36 patients with T2DM (44.4%). Among insulin-treated patients, 8 of 17 (47%) reported regular physical exercise, whereas 8 of 19 (42%) non-insulin-treated patients reported regular physical exercise (Table 1).

### 3.2. Cytokine Profile

#### 3.2.1. Effects of Glycemic Status and Physical Exercise Practice

Cytokines (IL-2, IL-4, IL-6, IL-10, IL-17, TNF-α, and IFN-γ) were evaluated in serum samples from non-diabetic (ND) and type 2 diabetes mellitus (T2DM) individuals according to physical exercise practice (Table 2). IL-2 concentrations were not significantly affected by either glycemic status or physical exercise practice.

Higher IL-4 and IL-6 concentrations were observed in physically active individuals in the T2DM group than in the respective ND group. Physical exercise modulated IL-10 concentrations differently by glycemic status, reducing IL-10 levels in ND individuals and increasing them in T2DM individuals. Moreover, sedentary T2DM individuals exhibited lower IL-10 concentrations compared with sedentary ND individuals. IL-17 concentrations were higher in physically active individuals, regardless of glycemic status, suggesting an exercise-related modulation of this cytokine. In contrast, TNF-α and IFN-γ concentrations were markedly elevated in physically active T2DM individuals compared with the other groups (Table 2).

#### 3.2.2. Effects of Glycemic, Physical Exercise, and Exogenous Insulin

**Figure 1:**
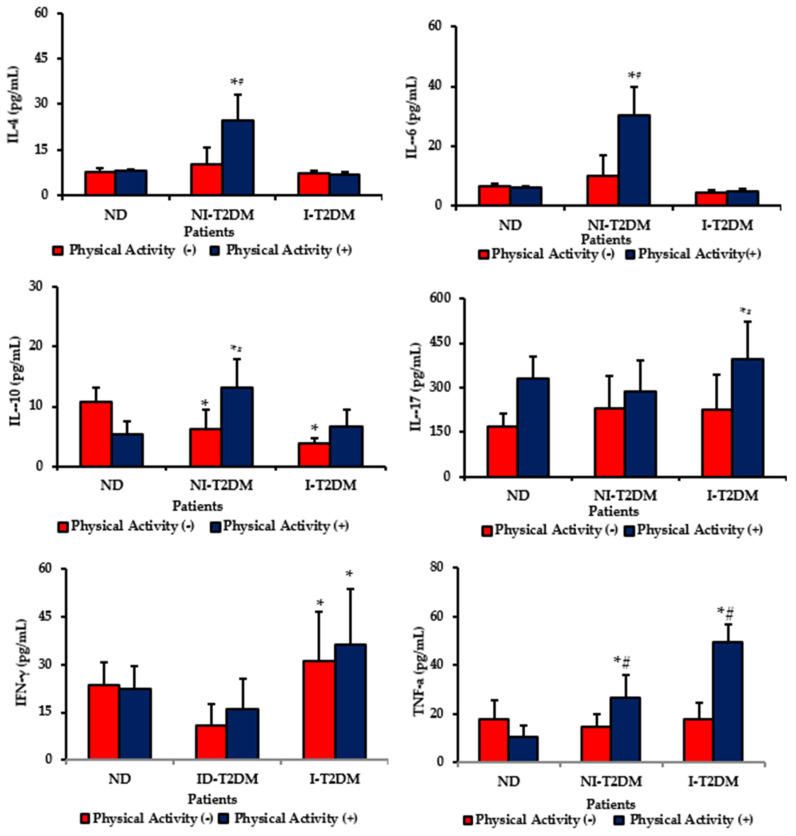
Cytokine concentrations (pg/mL) according to glycemic status and physical exercise practice in non-diabetic (ND), non-insulin-dependent type 2 diabetes mellitus (NI-T2DM), and insulin-dependent type 2 diabetes mellitus (I-T2DM) individuals. *p* < 0.05. * Indicates significant intergroup differences between control and diabetic individuals within the same physical exercise status. # Indicates significant differences according to physical exercise practice within the same glycemic status group.

We also investigated whether cytokine levels were associated with hyperglycemia, physical exercise, and exogenous insulin use. Physically active non-insulin-dependent type 2 diabetes mellitus (NI-T2DM) individuals exhibited higher IL-4 and IL-6 concentrations than sedentary NI-T2DM individuals and non-diabetic controls (Figure 1).

IL-10 concentrations were reduced in sedentary diabetic individuals regardless of insulin use. In contrast, physically active NI-T2DM individuals showed increased IL-10 levels. Furthermore, IL-17 concentrations were higher in physically active I-T2DM individuals than in sedentary I-T2DM individuals.

IFN-γ concentrations were elevated in I-T2DM individuals irrespective of physical exercise status. TNF-α concentrations were markedly increased in physically active insulin-treated T2DM (I-T2DM) individuals compared with sedentary I-T2DM individuals. Non-insulin-treated T2DM (NI-T2DM) individuals also exhibited higher TNF-α levels than non-diabetic controls. The highest TNF-α concentrations were observed in physically active I-T2DM individuals (Figure 1).

#### 3.2.3. Correlation Between Glycemia and Cytokine Levels According to Physical Exercise and Insulin Dependence

**Figure 2:**
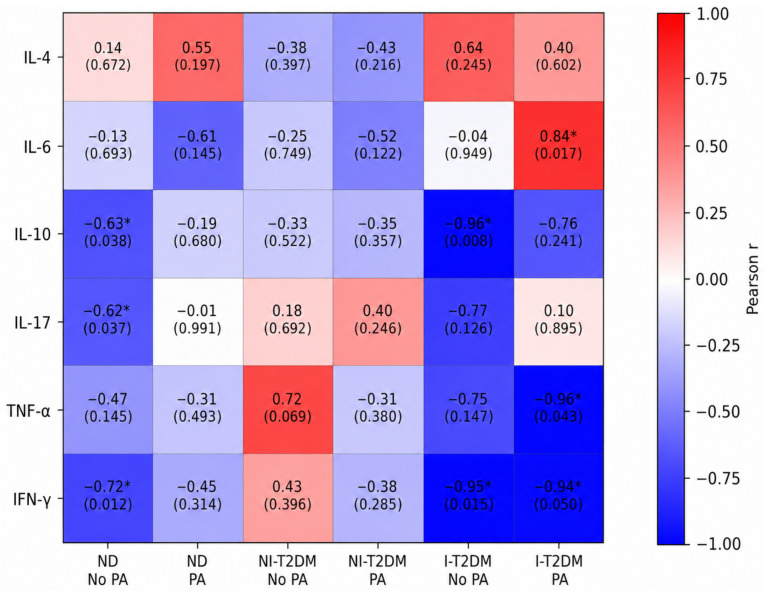
Heatmap showing Pearson’s correlation coefficients (r) between blood glucose levels (mg/dL) and serum cytokine concentrations (IL-4, IL-6, IL-10, IL-17, TNF-α, and IFN-γ; pg/mL) according to glycemic status and physical exercise practice. The columns represent non-diabetic individuals (ND), non-insulin-dependent type 2 diabetes mellitus individuals (NI-T2DM), and insulin-dependent type 2 diabetes mellitus individuals (I-T2DM), stratified according to the absence (No PA) or presence (PA) of physical exercise practice. Red tones indicate positive correlations, whereas blue tones indicate negative correlations. Color intensity is proportional to the magnitude of the correlation coefficient (r). Each cell displays the corresponding Pearson correlation coefficient and *p*-value. Significant correlations (*p* < 0.05) are indicated by an asterisk (*).

Cytokine concentrations and glycemia levels were analyzed using Pearson’s correlation test (Figure 2). The correlation analysis demonstrated distinct associations between blood glucose levels and cytokine concentrations across glycemic status, insulin dependence, and physical exercise. Among non-diabetic individuals without physical exercise practice, glucose levels showed significant negative correlations with IL-10 (r = −0.6302; *p* = 0.0376), IL-17 (r = −0.6220; *p* = 0.0368), and IFN-γ (r = −0.7209; *p* = 0.0123).

In individuals with non-insulin-dependent type 2 diabetes mellitus (NI-T2DM), no significant correlations were observed between glucose levels and cytokine concentrations, regardless of physical exercise. However, a trend toward a positive association between TNF-α and glucose levels was identified in sedentary NI-T2DM individuals (r = 0.7177; *p* = 0.0693).

In individuals with insulin-dependent type 2 diabetes mellitus (I-T2DM) who were not physically active, glucose levels were strongly and negatively correlated with IL-10 (r = −0.9648; *p* = 0.0079) and IFN-γ (r = −0.9466; *p* = 0.0147). In contrast, among physically active I-T2DM individuals, glucose levels showed a significant positive correlation with IL-6 (r = 0.8352; *p* = 0.0165), whereas significant negative correlations were observed with TNF-α (r = −0.9570; *p* = 0.0429) and IFN-γ (r = −0.9427; *p* = 0.0499).

**Figure 3:**
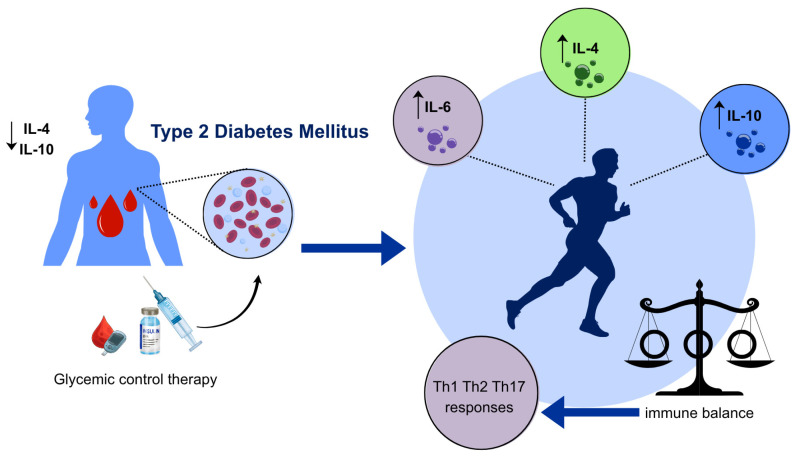
Physical exercise associated with glycemic control therapy promotes immunomodulation in type 2 diabetes mellitus by increasing IL-4, IL-6, and IL-10 levels, thereby helping maintain the Th1/Th2/Th17 balance and a more regulated inflammatory profile.

Figure 3 illustrates the proposed interaction between physical exercise and glycemic control therapy in patients with T2DM. The schematic representation summarizes the observed cytokine modulation, characterized by increased levels of IL-4, IL-6, and IL-10, which contribute to the maintenance of Th1/Th2/Th17 balance and a more regulated immune profile.

## 4. Discussion

The clinical and biochemical characteristics of diabetic patients depend on metabolic performance and immunological behavior in maintaining or not a proinflammatory state [28]. Inflammation can play a dual role in diabetes mellitus, as chronic inflammation promotes insulin resistance, and the hyperglycemic environment intensifies the response of proinflammatory pathways. Lifestyle modification modulates systemic inflammation and immunological parameters [29]. Changes in the immune system and alterations in serum concentrations of anti-inflammatory cytokines are associated with various exercise modalities, such as walking, cycling, and aerobics [23,30,31], resulting in improved insulin sensitivity and clinical improvement. In this study, we evaluated the impact of physical exercise and insulin therapy on the cytokine profile in patients with diabetes.

The alteration of the Th1/Th2 cytokine profile in type 2 diabetes mellitus (T2DM) has been associated with the exacerbation of the proinflammatory state due to an imbalance between anti-inflammatory and proinflammatory cytokines [32]. In this study, physical exercise increased both anti-inflammatory and proinflammatory cytokines in diabetic individuals. Interestingly, IL-10 was reduced in T2DM patients who did not exercise. IL-10 can regulate the innate immune response and Th1 and Th2 responses, restricting T cell activation and suppressing proinflammatory responses in tissues, leading to improved insulin sensitivity [33,34,35], suggesting that regular physical exercise is important for maintaining the balance between Th1 and Th2 cells and, consequently, an anti-inflammatory profile, in addition to improving glycemic levels in these patients. Interestingly, the increase in IL-4 and IL-6 concentrations associated with physical exercise was observed primarily in non-insulin-treated individuals with T2DM, suggesting a distinct cytokine response according to treatment status. Similarly, increased IL-4 concentrations were observed primarily in physically active non-insulin-treated individuals with T2DM. IL-4 is a key anti-inflammatory cytokine that regulates Th2 immune responses and has been associated with improved metabolic homeostasis and attenuation of chronic inflammation [9,10]. Thus, the elevated IL-4 levels observed in these individuals may further support the immunomodulatory benefits of regular physical exercise in T2DM.

In this study, physical exercise also increased IL-17 levels regardless of glycemic status. IL-17 is associated with a variety of inflammatory conditions and complications, and it appears to play a crucial role in insulin resistance and Type 2 Diabetes Mellitus (T2DM) [13,36]. Previous studies have demonstrated that elevated IL-17 levels contribute to chronic low-grade inflammation and metabolic dysfunction, both of which are key features of T2DM [14]. Furthermore, Th17 cells, which produce IL-17, have been implicated in the pathogenesis of certain autoimmune diseases, including rheumatoid arthritis, psoriasis, and multiple sclerosis, where they promote persistent inflammatory responses and tissue damage [15,37]. In the gastrointestinal tract, IL-17 plays a dual role: it supports epithelial barrier integrity and antimicrobial defense, yet it can also contribute to chronic intestinal inflammation when dysregulated, as seen in inflammatory bowel diseases [15,37]. It also functions in host defense against infectious diseases [13,38,39], particularly by recruiting and activating neutrophils, thereby reinforcing protection against extracellular bacterial and fungal pathogens [37]. Therefore, the exercise-induced increase in IL-17 observed in the present study may represent a complex immunomodulatory adaptation rather than merely an inflammatory response. These findings suggest that physical exercise, independent of hyperglycemia, can help maintain immune competence and host defense while modulating inflammatory pathways implicated in the pathophysiology of T2DM [40,41].

Previous studies have reported reductions in circulating TNF-α following exercise interventions in patients with type 2 diabetes, our findings demonstrated higher serum TNF-α and IFN-γ concentrations among physically active diabetic individuals [42,43]. These results should be interpreted in the context of the complex immunological adaptations induced by exercise, which vary according to training characteristics, disease status, and therapeutic management. Therefore, the observed cytokine profile may reflect an exercise-associated modulation of immune responses rather than an adverse inflammatory effect [44,45,46].

There is growing concern regarding therapeutic approaches for treating diabetic patients due to the potential progression of the disease with various clinical complications [47]. In this study, we also investigated whether the combined effects of hyperglycemia, physical exercise, and exogenous insulin influenced changes in cytokine levels. Overall, anti-inflammatory cytokines (IL-4 and IL-10) were higher in non-insulin-dependent patients with diabetes mellitus who exercised, suggesting that physical exercise is essential for modifying the typical proinflammatory profile in these patients’ blood.

Although physical inactivity is considered a proinflammatory risk factor [48], the highest TNF-α concentrations in the present study were observed in physically active insulin-treated T2DM individuals. TNF-α is known to impair insulin signaling and contribute to insulin resistance in skeletal muscle cells [49,50]. However, physical exercise promotes complex immunometabolic adaptations and can modulate the production of both proinflammatory and anti-inflammatory cytokines [30]. Therefore, the elevated TNF-α levels observed in physically active insulin-treated individuals can reflect an exercise-associated immunomodulatory response rather than an exclusively detrimental inflammatory state.

IFN-γ levels increased in the serum of patients with diabetes mellitus, insulin-dependent, regardless of physical exercise. These data suggest that physical exercise, such as walking, was insufficient to modify the typical proinflammatory profile in diabetic patients. Further studies should be conducted using other types of physical exercise in insulin-dependent diabetic patients who present with more clinical complications.

Furthermore, some cytokine levels correlated with blood glucose concentrations. Notably, the strongest associations were observed in insulin-dependent diabetic patients. In this population, blood glucose levels showed strong correlations with both anti-inflammatory and proinflammatory cytokines, including IL-10, TNF-α, and IFN-γ, suggesting that glycemic control may play a central role in shaping immune responses in individuals with more advanced metabolic impairment. These findings reinforce the close interaction between metabolic regulation and immune function in type 2 diabetes mellitus.

Among insulin-dependent diabetic patients who practiced physical exercise, blood glucose levels were positively correlated with IL-6 and negatively correlated with TNF-α and IFN-γ. Studies demonstrate that IL-6 is involved in the anti-inflammatory and metabolic adaptations associated with regular physical exercise in individuals with metabolic diseases [51,52,53]. In addition, IL-6 can be synthesized and released by skeletal muscle during exercise, acting as a myokine that improves insulin sensitivity and metabolic regulation [51,54,55,56].

IL-6 can inhibit circulating TNF-α levels in healthy individuals by modulating TNF-α release [57,58]. Also, IL-6 release during exercise increases the production and release of other anti-inflammatory cytokines, such as IL-10 [59,60]. This positive correlation between glucose levels and IL-6 may be important for diabetic patients, especially those who are insulin-dependent, in whom glycemic levels are higher, since IL-6 increases lipolysis and fat oxidation in adipose tissue during exercise and increases insulin sensitivity in patients with type 2 diabetes [55,58,60].

The differences observed between individuals treated with insulin and those not treated suggest that insulin therapy may affect cytokine responses in patients with type 2 diabetes mellitus (T2DM). In this study, distinct patterns were identified for cytokines such as IL-4, IL-6, TNF-α, and IFN-γ across treatment groups. This implies that both metabolic control and therapeutic interventions can influence inflammatory and immune responses. These findings highlight the importance of considering insulin therapy when assessing cytokine profiles and the immunomodulatory effects of physical exercise in T2DM.

A limitation of this study is the relatively small sample size in certain subgroups, particularly after stratification by glycemic status, insulin therapy, and physical activity levels. It was also not possible to conduct subgroup analyses due to comorbidities such as hypertension; therefore, we could not assess how these conditions might have influenced cytokine profiles and exercise responses. Additionally, although participants with type 2 diabetes mellitus (T2DM) received dietary recommendations as part of their standard clinical management, we did not formally monitor dietary intake during the study. Consequently, it was not possible to assess how individual dietary variations might have impacted glycemic control and cytokine levels. Future studies with larger cohorts are needed to confirm and expand upon these findings, as well as to evaluate other potentially associated parameters.

## 5. Conclusions

In conclusion, the present findings demonstrate that physical exercise, combined with appropriate glycemic control therapy, is associated with the maintenance of balance among Th1, Th2, and Th17 cytokine profiles in patients with type 2 diabetes mellitus. The observed cytokine modulation suggests an anti-inflammatory profile among physically active individuals, reinforcing the importance of exercise as an adjunct strategy in diabetes management. These findings further support the involvement of immune regulatory mechanisms in the adaptations associated with glycemic control and physical exercise.

## Figures and Tables

**Table 1:** Clinical characteristics of individuals according to glycemic status: normoglycemic individuals (ND) and patients with type 2 diabetes mellitus (T2DM).

Individuals	ND	T2DM
Number	15	36
Age (years)	27.2 ± 2.5	38.7 ± 4.6
Gender (% female)	6/15 (40%)	15/36 (41.7%)
Body Mass Index (kg/m^2^)	21.3 ± 0.6	24.2 ± 4.7
Glucose (mg/dL)	78.0 ± 6.4	234.0 ± 91.5 *
HbA1c (%)	5.0 ± 0.4	6.6 ± 0.9 *
Insulin dependent	------	47% (17)
Hypertension	0/15 (0%)	5/36 (13.8%)
Practice of Physical Exercise	7/15 (46.7%)	16/36 (44.4%)
Practice of Physical Exercise for an Insulin-Dependent Person	------	8/17 (47%)
Practice of Physical Exercise in Non-Insulin-Dependent	------	8/19 (42%)

Notes: HbA1c—hemoglobina glicada. * *p* < 0.05 vs. ND group.

**Table 2:** Serum cytokine concentrations (pg/mL) according to physical exercise practice and glycemic status in individuals with normoglycemia (ND) and type 2 diabetes mellitus (T2DM).

Groups	ND		T2DM	
Physical Exercise	No	Yes	No	Yes
IL-2	18.4 ± 3.9	20.4 ± 6.7	18.9 ± 7.5	25.6 ± 5.4
IL-4	8.0 ± 0.5	7.9 ± 1.0	9.4 ± 3.7	18.3 ± 5.3 *
IL-6	6.5 ± 2.2	6.0 ± 0.8	8.1 ± 5.5	17.6 ± 6.9 *
IL-10	10.8 ± 2.4	5.3 ± 2.1 ^#^	5.2 ± 2.3 *	11.3 ± 2.3 *^#^
IL-17	169.2 ± 43.1	331.8 ± 74.2 ^#^	293.3 ± 112.9	359.1 ± 93.2^#^
TNF-α	17.6 ± 7.7	10.6 ± 4.9	25.7 ± 3.1	82.9 ± 10.8 *^#^
IFN-γ	23.5 ± 7.4	22.3 ± 7.6	17.8 ± 2.2	34.3 ± 9.8 ^#^

*p* < 0.05. * Indicates significant intergroup differences between control and diabetic individuals within the same physical exercise status. ^#^ Indicates significant differences according to physical exercise practice within the same glycemic status group.

## Data Availability

The authors will make the data supporting the study’s interpretations available upon request.

## Abbreviations

ADA	American Diabetes Association
ANOVA	Analysis of Variance
BMI	Body Mass Index
CBA	Cytometric Bead Array
CRP	C-Reactive Protein
DM	Diabetes Mellitus
GI	Glycemic Index
HIV	Human Immunodeficiency Virus
I-T2DM	Insulin-Dependent Type 2 Diabetes Mellitus
IFN-γ	Interferon Gamma
IL	Interleukin
ND	non-diabetic
NI-T2DM	Non-Insulin-Dependent Type 2 Diabetes Mellitus
SD	Standard Deviation
T2DM	Type 2 Diabetes Mellitus
Th	T Helper
TNF-α	Tumor Necrosis Factor Alpha
